# Foliar application of microbial and plant based biostimulants increases growth and potassium uptake in almond (*Prunus dulcis* [Mill.] D. A. Webb)

**DOI:** 10.3389/fpls.2015.00087

**Published:** 2015-02-23

**Authors:** Sebastian Saa, Andres Olivos-Del Rio, Sebastian Castro, Patrick H. Brown

**Affiliations:** ^1^Escuela de Agronomía, Pontificia Universidad Católica de ValparaísoQuillota, Chile; ^2^Department of Plant Sciences, University of California, DavisDavis, CA, USA

**Keywords:** foliar fertilizer, rubidium, almond, microbial fermentation, *Ascophyllum nodosum*, GroZyme, MegaFol

## Abstract

The use of biostimulants has become a common practice in agriculture. However, there is little peer-reviewed research on this topic. In this study we tested, under controlled and replicated conditions, the effect of one biostimulant derived from seaweed extraction (Bio-1) and another biostimulant derived from microbial fermentation (Bio-2). This experiment utilized 2-years-old almond plants over two growing seasons in a randomized complete design with a full 2 × 4 factorial structure with two soil potassium treatments (125 μg g^-1^ of K vs. 5 μg g^-1^) and four foliar treatments (No spray, Foliar-K, Bio-1, Bio-2). Rubidium was utilized as a surrogate for short-term potassium uptake and plant growth, nutrient concentration, and final plant biomass were evaluated. There was a substantial positive effect of both biostimulant treatments on total shoot leaf area, and significant increases in shoot length and biomass under adequate soil potassium supply with a positive effect of Bio-1 only under low K supply. Rubidium uptake was increased by Bio-1 application an effect that was greater under the low soil K treatment. Though significant beneficial effects of the biostimulants used on plant growth were observed, it is not possible to determine the mode of action of these materials. The results presented here illustrate the promise and complexity of research involving biostimulants.

## INTRODUCTION

The use of biostimulants, defined here as ‘a substance or material, with the exception of nutrients and pesticides, which has the capacity to beneficially modify plant growth’ has grown dramatically over the past decade and it is predicted that the market for biostimulants will exceed US$2 billion by the year 2018 (Saa-Silva et al., 2013; Calvo et al., 2014). Agricultural biostimulants are derived from a wide range of materials including but not limited to, living microbial cultures; extracts of microbial, animal or plant origin; soil organic residues (humates, fulvates); industrial by-products and chemicals, and synthetic molecules. The mode of action of the majority of biostimulants is poorly or not understood and has been variously ascribed to hormone composition, the presence of plant signaling molecules or the presence of molecules that facilitate the transport and efficacy of mineral nutrients (Saa-Silva et al., 2013; Calvo et al., 2014). In a majority of cases the specific metabolic components of the biostimulant have not been characterized and hence the function is unknown. Determining the function of biostimulants is made more difficult since many of these products contain naturally occurring or commercially added micronutrients, sugars, amino acids and other compounds that may have synergistic, complementary or no effects or may have been added merely for marketing or commercial registration purposes. Separating the effect of the one or more active ingredients from the host of additional components is often very difficult.

While there is a large body of applied field trials demonstrating the benefits of diverse biostimulants on plant growth (Saa-Silva et al., 2013; Calvo et al., 2014) there is also a great deal of inconsistency in response likely due to variations in species and environment under which the field trials were conducted and a lack of understanding of the specific metabolic function of the biostimulant being applied. In recent years advances in understanding of several commercially available biostimulants has been made possible through the application of phenomic and molecular approaches (Sharma et al., 2012, 2014; Jannin et al., 2013; Saa-Silva et al., 2013; Wargent et al., 2013; Billard et al., 2014; Petrozza et al., 2014). Results of these studies suggest that biostimulants based upon plant extracts or microbial cultures may contain metabolites involved in stress perception that can act to ‘prime’ plants (Conrath et al., 2006) to better resist future biotic or abiotic stresses. Once a plant has perceived a stress, biostimulants have also been shown to enhance plant stress tolerance mechanisms (Petrozza et al., 2014). The ability to adapt to stresses and to upregulate stress tolerance mechanisms may help diminish the growth suppression that would typically occur when a stress is perceived. The study of plant stress signaling and response molecules is one of the most active areas of plant research and has resulted in the identification of many small molecules that play critical roles in stress signaling and plant response (Zhu, 2002; Chaves et al., 2003; Mittler, 2006; Albacete et al., 2014; Delorge et al., 2014; Golldack et al., 2014; Lastdrager et al., 2014; Pottosin and Shabala, 2014; Shi and Chan, 2014). Discoveries in this field hold great promise for the identification of the mode of action of current biostimulants and for the development of biostimulants that target specific metabolic pathways and physiological responses.

The two classes of biostimulants that have been researched in greatest detail are those derived from seaweeds (Castro et al., 2012; Jannin et al., 2013; Stirk et al., 2013, 2014; Billard et al., 2014; Petrozza et al., 2014) and those containing live microbial cultures or products derived from microbial cultures (Chen et al., 2002, 2003; Wargent et al., 2013; Calvo et al., 2014; Lucas et al., 2014; Niazi et al., 2014; Timmusk et al., 2014). Several commercial biostimulant products in this realm also incorporate into their products, amino acids, betaines, and plant vitamins known to be involved in plant stress signaling and response processes (Knight and Knight, 2001; Zhu, 2002; Chaves et al., 2003; Lastdrager et al., 2014). In addition to their effects on plant stress tolerance, biostimulants based upon seaweeds and microbial cultures and extracts may stimulate nutrient uptake and translocation (Paradikovic et al., 2011; Dodd and Perez-Alfocea, 2012; Jannin et al., 2013; MacDonald et al., 2013; Calvo et al., 2014; Sabir et al., 2014).

While much of the research on the use of biostimulants has focused on their benefits under stress conditions, there are many reports of growth stimulation of biostimulants when plants are grown under conditions where stress was minimized (Saa-Silva et al., 2013; Calvo et al., 2014). Whether the mode of action of a biostimulant is similar under stress and non-stress conditions, is unknown.

In the research described here, two biostimulant formulations, representative of plant based extracts (MegaFol, Valagro, Atessa, Italy) and microbial extracts (GroZyme, Ag Spectrum, DeWitt, IA, USA) was examined. The MegaFol formulation is derived from seaweed (*Ascophyllum nodosum*) with the addition of amino acids (proline and tryptophan), sugars (glycosides, polysaccharides), vitamins and betaines that have been identified as stress signaling and response molecules in other studies (Atkinson et al., 2013; Kissoudis et al., 2014; Minocha et al., 2014). Recent evidence suggests that MegaFol upregulates a number of stress response pathways in tomato (Petrozza et al., 2014). GroZyme is a non-living extract derived from microbial fermentation of grain utilizing plant growth promoting rhizobacteria and other bacteria. Applied to the soil GroZyme enhances organic nitrogen transformations (Chen et al., 2002, 2003). Recently GroZyme has been shown to stimulate plant growth when applied as a foliar application and to result in increased leaf potassium in corn (Ag Spectrum, unpublished field trials) and greater translocation of foliar applied Zn (Tian et al., 2015). The mode of action of foliar GroZyme is unknown.

The mechanisms by which foliar applied materials penetrate the leaf surface is complex and will be influenced by size and polar nature of the applied molecules (Fernandez and Brown, 2013). The first constraint to leaf penetration is the negatively charged, hydrophobic cuticular layer which limits the penetration of positive ions and hydrophilic molecules (Fernandez and Brown, 2013). The cuticle also contains stomata and trichomes, which may also function as a pathway for small molecular weight compounds (Eichert et al., 2008). The apoplastic space in leaves is dominated by negatively charged exchange sites which may interact with positively charged molecules, restricting their movement. The metabolism of foliar absorbed materials in the leaf apoplast is poorly understood, but depending upon the composition and concentration of the applied materials may influence plant metabolism directly through the supply of nutrients, metabolites or molecules that correct nutrient deficiencies or alter metabolic pathways or indirectly through short-term effects on cellular pH or electrochemical balance.

Biostimulants are widely used in many agricultural practices, particularly high value vegetable and fruit tree production systems and yet little is known of their efficacy or mode of action. Predicting plant response to the application of biostimulants is complex due both to the uncertainty surrounding the foliar absorption and the lack of knowledge of the mode of action of these products. This work aims to document plant growth response under carefully controlled conditions and to examine the impact of biostimulant applications on the uptake of Rb^+^ (as a K^+^ analog) under potassium replete and deficient conditions.

## MATERIALS AND METHODS

### EXPERIMENT SETUP AND TREATMENT APPLICATION

Forty dormant 1-year-old almond trees var. ‘Nonpareil’ grafted to ‘Nemaguard’ rootstock were purchased from a commercial CA nursery in winter 2011. Plants were selected for uniformity and then roots and shoots were further trimmed to ensure uniformity of size prior to planting into 20 l pots and randomly allocated to the treatment groups. Pots were filled with virgin fritted illitic clay combining two commercial products in a ratio of 1–3 (commercial names “Turface MVP” and “Turface Profile Greens Grade Natural,” Turface Athletics, Buffalo Grove, IL, USA) and grown under natural light conditions in a temperature-controlled greenhouse maintained between 24 and 26°C, 40% relative humidity (RH; daytime) and 18–20°C, 80% RH (Nighttime). The potting media was selected because of its low native nutrient content, neutral pH (6.8), low density (0.56), high water holding capacity and good porosity (0.77), and prior experience that it is an excellent media for almond growth. Due to its high porosity this growth media can be watered abundantly with no risk of root anoxia. Daily plant water use was determined by frequent weighing and irrigation was provided on demand to maintain plants at near field capacity at all times. Stem water potential readings using a pressure chamber (Moisture Equipment Corp., Santa Barbara, CA, USA) were taken periodically to monitor plant water status throughout the experiment. Twenty plants were irrigated with half strength Hoagland solution during each irrigation event, while the other 20 plants received the same solution, but with reduced in potassium concentration (125 μg g^-1^ of K vs. 5 μg g^-1^). Growth of trees was vigorous and control trees had reached 2 m height with extensive branching and dense canopies by fall 2011. By the middle of summer 2011, low potassium treated plants had developed visual signs of moderate K deficiency symptoms (marginal leaf tip chlorosis in older leaves). This was confirmed by leaf tissue analysis in which low K trees had a significantly lower K concentration of 2.1% and adequate K trees had a K concentration of 2.5% at the beginning of summer 2011 (analytical methods described below).

In addition, individual whole plant pictures taken at the end of summer were analyzed using ImageJ program (ImageJ, U. S. National Institutes of Health, Bethesda, MD, USA) to confirm potassium effects on tree size. All plant pictures were taken at the same position and distance with a white background. Then, an automatized ImageJ script to analyze leaf area developed by Saa and Brown (2014) was used. Control plants had on average 45% more leaf area than low K plants by the end of the 2011 growing season. Plants entered dormancy in November of 2011 and in January 2012, plants were moved to a larger outdoor, screened greenhouse under natural temperature and light conditions and all plants were pruned back to a uniform canopy size and selecting four branches per plant. Soil K treatments established in 2011 were continued for the duration of the experiment.

### FOLIAR TREATMENTS

Four foliar treatments consisting of two biostimulant formulations, one foliar K treatment (described below) and one control treatment (no spray) were utilized. No surfactant was added in any treatment. Plants that received the two biostimulant treatments were sprayed two times in summer of 2011 at 7 days intervals (160 and 167 days after full bloom) and three times in spring 2012 at 7 days intervals (67, 74, and 81 days after full bloom). Sprays were applied between 10:00 and 11:30 am. Environmental temperatures and RH percentage for 2012 applications were between 20 and 24°C and 20–50% RH, respectively. Foliar potassium was sprayed three times in spring 2012, but no spray application was performed in 2011. Treatment application was done using a hand sprayer until all leaf surfaces were wetted; care was taken to ensure no direct soil application occurred.

The two biostimulant products utilized in these experiments were based upon commercial products currently in widespread use in the USA and for which positive results have been reported in field use in almonds in California. Both products are proprietary industrial extractions from plant and microbial feedstocks and contain a wide range of known and unknown functional components and low levels of plant nutrients. The full mechanism of their biological activity is unknown.

Biostimulant Product 1 was a mixture of three products manufactured by Valagro, SpA (Atessa, Italy) under the trade names MegaFol, Brexil-Zn, and MC-Extra. MegaFol is a mixture of amino acids (proline and tryptophan), glycosides, vitamins, polysaccharides, betaines, organic nitrogen, and carbon derived from *A. nodosum* and other plant materials (Paradikovic et al., 2011; Petrozza et al., 2014). MC-Extra is derived from the seaweed *A. nodosum* and contains mannitol, cytokinins, and betaines. Brexil-Zn is a lignosulfonate based Zn formulation. Biostimulant product 1 contained small amounts of N, K, and Zn (**Table 1**).

**Table 1:** Composition of treatments.

Treatments	Formulation	Element	Concentration of nutrient in final solution (μg g^--1^)	Times applied(Sprays)	Period of application^1^
Soil –K adequate	Half Hoagland	All essential elements in adequate dosage	K at 125	N.A.	At each irrigation event
Soil – K deficit	Half Hoagland	All essential elements in adequate dosage (except K)	K at 5	N.A.	At each irrigation event
Foliar – control	No-application	None	0	0	None
Foliar – (Bio-1)	Multi-element Mix Plus Biostimulant^a^	N-K-Zn	132-581-119	5	Summer 2011 Spring 2012
Foliar – (Bio-2)	Multi-element Mix Plus Biostimulant^b^	N, P, K, S, Cu, Fe, Mn, Zn, GroZyme	6964, 7218, 2168, 88, 22, 44, 48, 88, 544	5	Summer 2011 Spring 2012
Foliar – K	K_2_O	K	581	3	Spring 2012

*^1^Bio-1 and Bio-2 were applied two times in summer 2011(at 160 and 167 days after full bloom) and three times in spring 2012 (67, 74, and 81 days after full bloom)*.

*^a^A mixture of three products manufactured by Valagro, SpA (Atessa, Italy) under the trade names MegaFol, Brexil-Zn, and MC-Extra. MegaFol is a mixture of amino acids (proline and tryptophan), glycosides, vitamins, polysaccharides, betaines, organic nitrogen and carbon derived from *Ascophyllum nodosum* and other plant materials (Paradikovic et al., 2011; Petrozza et al., 2014). MC-Extra is derived from the seaweed *A. nodosum* and contains mannitol, cytokinins, and betaines. Brexil-Zn is a lignosulfonate based Zn formulation*.

*^b^Biostimulant manufactured by Ag Spectrum, DeWitt, IA, USA under the trade name GroZyme and is a microbial fermentation product derived from a proprietary mix of organic cereal grains inoculated with specific bacterial cultures and fermented*.

Biostimulant Product 2 is manufactured by Ag Spectrum, DeWitt, IA, USA under the trade name GroZyme and is a microbial fermentation product derived from a proprietary mix of organic cereal grains inoculated with specific bacterial cultures and fermented. The fermentation process occurs under controlled environmental conditions until a specific metabolic profile is achieved at which time the live bacterium is lysed and the material is filtered to remove large particles. This concentrate is then extended and stabilized to make the final product. The metabolic basis for the biological activity of GroZyme is not known, however, field observations suggest that GroZyme functions to enhance plant growth by enhancing K metabolism and sugar transport (Ag Spectrum, unpublished results). To replicate typical field practice, small amounts of two additional proprietary inorganic Ag Spectrum products, CleanStart (ammonium hydroxide, urea, orthophosphoric acid) and Kickoff (micronutrient mix of Fe, Mn, Cu, Zn predominantly derived from nitrate sources with additional surfactants and stabilizers) were included in this biostimulant treatment.

The third foliar treatment was Manniplex K (Brandt, Springfield, IL, USA), derived from potassium carbonate with additional mannitol and was provided at the same K concentration as that found in Biostimulant 1 (**Table 1**).

A control treatment consisting of no spray application was also included. Constructing control treatments to distinguish the single functional components of complex mixtures such as biostimulants 1 and 2 used here, is both impractical and not likely to be instructive. To minimize the possibility that the observed responses to the biostimulant product were a result of the provision of the included mineral nutrients we maintained all plants with abundant levels of all essential plant nutrients through soil application (with the exception of the low K soil treatment). Visual observation and leaf tissue analysis verified that all K^+^ treatments received luxury levels of all essential elements suggesting that the responses observed in these experiments were not the result of the alleviation of a plant nutrient deficit. A full description of all treatments is included in **Table 1**.

### RUBIDIUM TREATMENTS

As potassium has no readily usable radioactive or stable isotope, rubidium has long been used as a tracer for short-term potassium uptake studies (Pettersson and Jensen, 1979; Reickenberg and Pritts, 1996; Restrepo-Diaz et al., 2008). Following the third round of foliar spray application in 2012, all plants were irrigated with deionized water at 3x pore volume then irrigated with no additional K for 10 days to reduce K present in the media. Following this K wash out period all plants were irrigated for 7-days with a half strength Hoagland solution containing 70 μg g^-1^ of rubidium (Rb) and no potassium. After the 7-days treatment leaves were sampled for Rb determination as described below, then plants were returned to their original K treatments for the remainder of the 2012 season.

### PLANT GROWTH AND NUTRIENT MEASUREMENTS

Two well-lit shoots (subsamples) from opposite sides of the outer canopy of each plant were randomly selected and marked in March 2012 and used to determine shoot extension and leaf length on five occasions over the growing season at 15 days intervals commencing April 20th, 2012 (53 days after full bloom). The first two measurements were performed prior to the foliar applications of 2012, while the last three measurements were performed during (third) and after (fourth and fifth) the foliar sprays in 2012. The number and length of all leaves as well as the internode length of each shoot were recorded at each sampling date. To determine the relationship between leaf length and leaf area, a subsample of leaves were randomly collected from each plant (excluding the shoots used for growth determination) and individual leaf size, length, and area were determined using a portable scanner (CanoScan LIDE110, Canon Corporation, Japan) and an image analysis program (ImageJ, U. S. National Institutes of Health, Bethesda, MD, USA). This data was then used to construct a regression model to predict leaf area from leaf length (*r*^2^ = 0.92) throughout the season.

Leaf nutrient concentration (Rb and K) was determined 2 weeks after Rb application. In each plant, young (developing leaves) and mature (leaves that had reached final size) leaves were sampled independently resulting for a total of 80 samples. Samples were dried at 65°C until a constant weight was reached and then ground using a Wiley mill to pass through a 40-mesh screen, digested in a microwave digestion system and sent to the UC Davis analytical lab for the analysis of Rb and K by inductively coupled plasma atomic emission spectroscopy (ICP-590; Saa et al., 2014). All plants were then harvested at the completion of the experiment during tree dormancy in winter of 2013 and trunk diameter (0.7 m above the rootstock), weight of the primary scaffolds, and weight of the two and 1-year-old shoots (2011 and 2012 shoots) was determined.

### STATISTICAL ANALYSIS

The experimental design was a randomized complete design with a full factorial structure. Soil potassium treatments were 5 μg g^-1^ of K and 125 μg g^-1^ of K in each irrigation event. Foliar treatments were (i) control (Foliar-Control); (ii) Biostimulant 1 (Bio-1); (iii) Biostimulant 2 (Bio-2); and (iv) Foliar-K. Growth measurements were analyzed for statistical significance using sampling date as a main plot and running a repeated measurement analysis in the JMP program version 11, SAS Institute Inc., Cary, NC, USA, 1989–2013. Finally, all selected outputs were plotted using Sigma Plot program version 12.5, Systat Software, Inc., San Jose, CA 95110, USA.

## RESULTS

### SHOOT DEVELOPMENT

**FIGURE 1:**
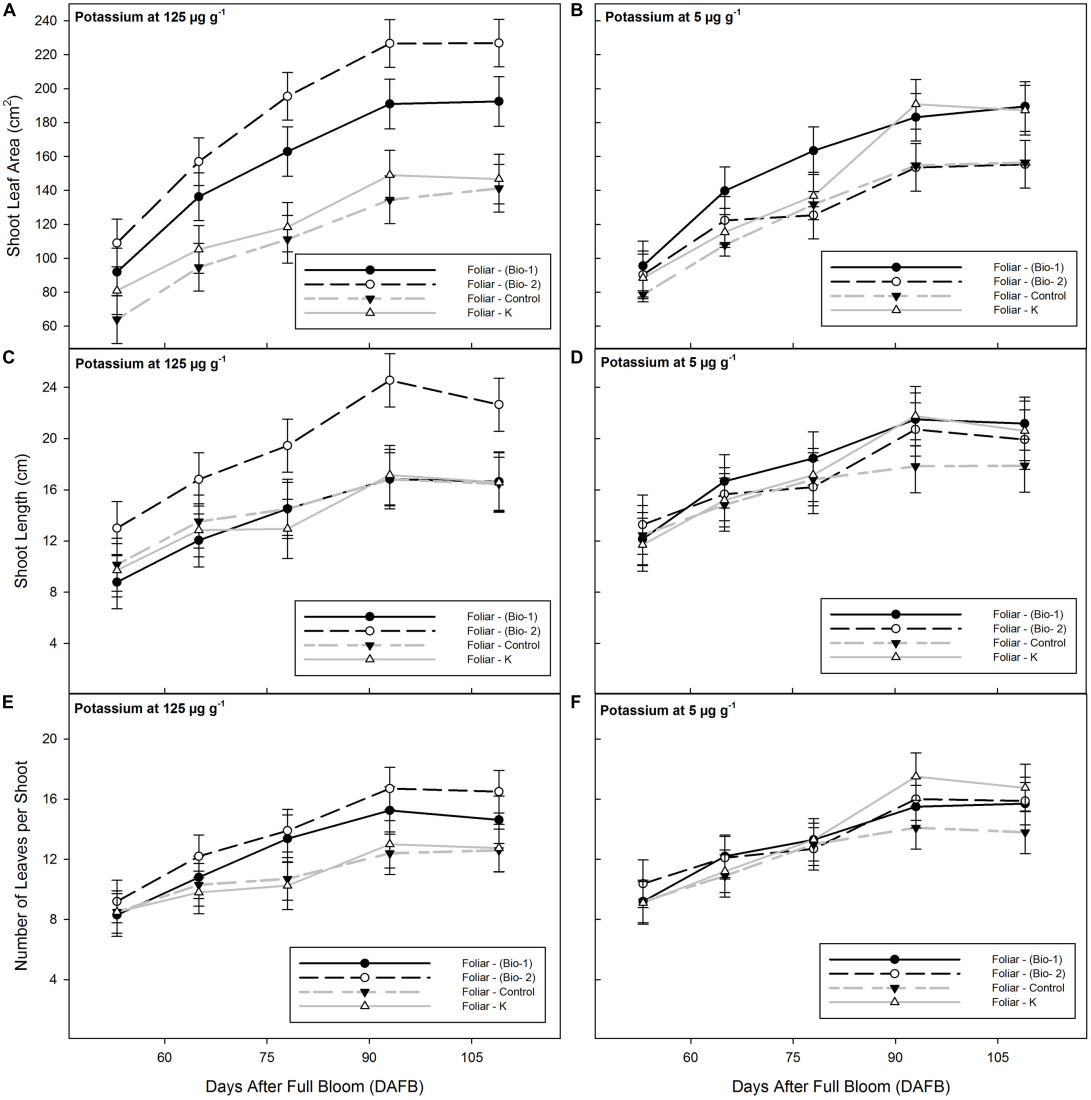
**Shoot leaf area **(A,B)**, shoot length **(C,D)** and number of leaves **(E,F)** of 2-years-old almond plants grown with adequate (124 ppm), or insufficient K (5 ppm) and treated with three foliar applications of either biostimulant mixture 1 (Bio-1), biostimulant Mixture 2 (Bio-2), foliar K supplementation (Foliar-K), or water sprayed controls (Foliar Control).** See **Table 1** for treatment details.

Total shoot leaf area was significantly affected by sampling date, foliar spray, and a soil K × foliar interaction. Shoot leaf area was significantly increased by the application of Bio-2 in contrast to control or Foliar K treated plants at all sample dates under the adequate K regime (125 μg g^-1^ K), but there was no a significant effect under 5 μg g^-1^ (**Figures 1A,B**). By the last sample date shoot leaf area on the Bio-2 plants was 195% greater than in control plants. This effect was a result of significant increases in shoot length and an increase in number of leaves per shoot and by an increase in size of individual leaves (**Figures 1A,C,E**).

Bio-1 improved shoot leaf area in comparison with the control irrespective of the soil potassium treatment at each of the four final sample dates (125 μg g^-1^ K or 5 μg g^-1^). The total increment in shoot leaf area was 160% greater than controls (**Figure 1A,B**). The increment in shoot leaf area with Bio-1 was due to a small increment in total leaf number per shoot and a significant increment in average leaf size.

Foliar-K had no effect on any plant growth parameter in plants grown with adequate K (125 μg g^-1^). Under low K conditions (5 μg g^-1^), at the fourth and fifth sampling dates, Foliar K resulted in a significant increase in shoot leaf area and a positive but smaller increase in shoot length and leaf number (**Figures 1A,C,E**). The significant increment in leaf area was observed from 78 to 93 days after full bloom in plants treated with 5 μg g^-1^ K (**Figure 1**). Foliar K treatments were applied immediately after sampling date 2 and the increment in growth likely reflects and partial alleviation of K deficiency in the low K treated trees.

Under 125 μg g^-1^ K, Bio-2 was the only treatment that significantly improved shoot length. Final shoot length averaged 23 cm in the Bio-2 treatment, and 16 cm in the remaining treatments. Under 5 μg g^-1^ K, control plants had smaller shoots than the sprayed plants by the last sampling date. Shoot length increased from 53 to 93 days after full bloom, but no further increment was detected after this date.

The number of leaves per shoot was higher in the biostimulant treatments than in the control plants, irrespective of the soil K conditions with the greatest difference seen at 125 μg g^-1^ K. Across the different soil K conditions there was an average of eight leaves at 53 days after full bloom vs. 12 leaves at 109 days after full bloom.

### RUBIDIUM UPTAKE

**FIGURE 2:**
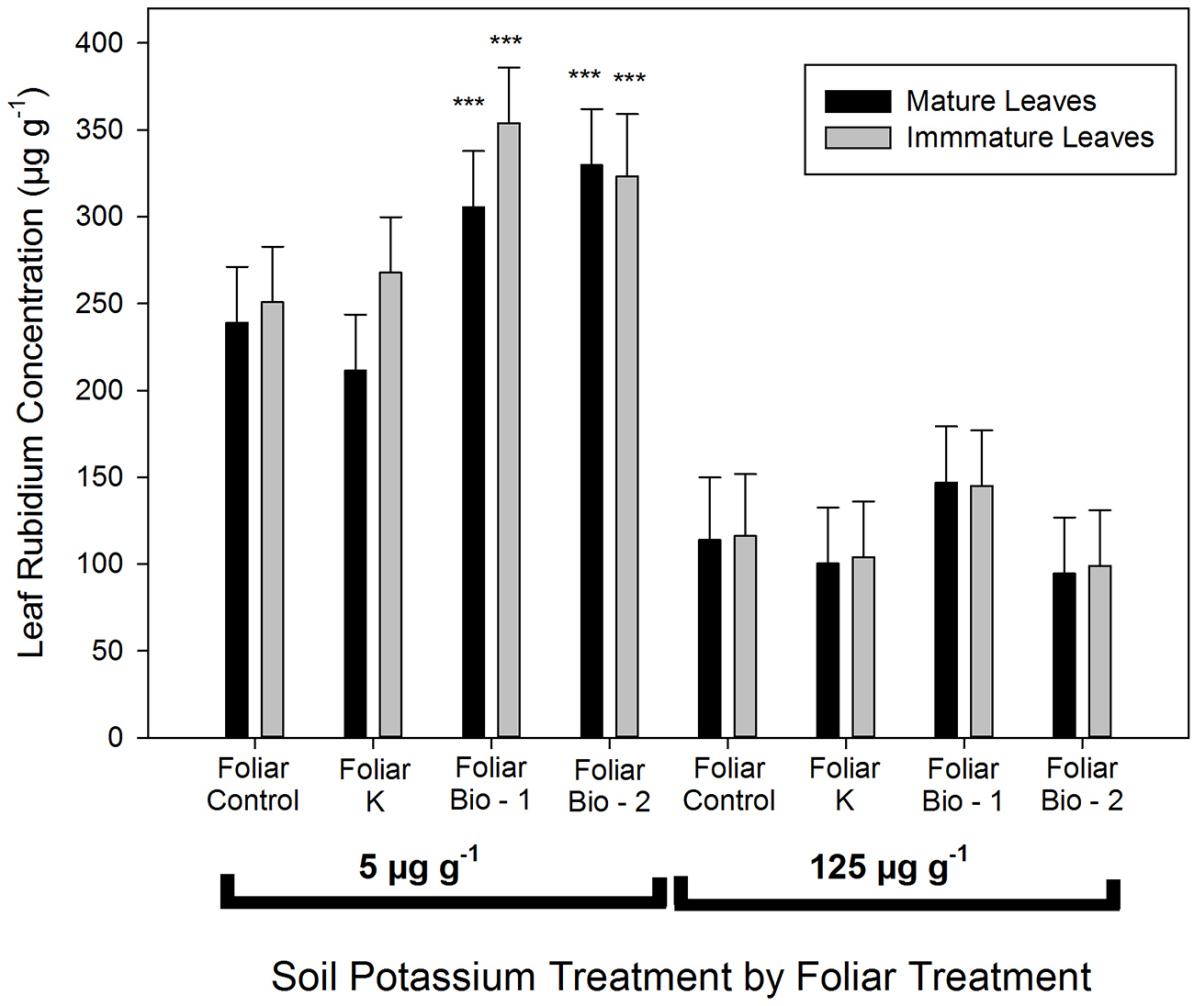
**Leaf rubidium concentration in mature (leaves that had reached final size) and immature (developing leaves) leaves of almond plants following a 7 days Rb uptake period in plants precultured in nutrient media provided with 5 or 124 μg g^**–1**^.** Multiple mean comparison was performed using Dunnett’s test choosing foliar control plants as the control level at α = 0.05, *n* = 20. This analysis was performed separately for plants provided with 5 and 124 μg g^-1^. Asterisks denote significant differences between foliar treatments among the same soil potassium treatment.

Biostimulant treatments significantly enhanced Rb uptake from the soil in the 5 μg g^-1^ K treatment (**Figure 2**). This effect was consistent in both leaf types (mature and immature leaves). Control plants and foliar K treated plants with low soil K supply (5 μg g^-1^ K) had average leaf concentrations of 238 μg g^-1^, while plants from Bio-1 and Bio-2 had 305 and 330 μg g^-1^, respectively. There was a slight, but non-significant reduction in leaf Rb in plants receiving foliar K applications when contrasted with non-sprayed control leaves. Plants grown in 5 μg g^-1^ K had a threefold higher final Rb concentration when compared with plants grown in 125 μg g^-1^ K, however, there were no significant differences in Rb concentration due to foliar sprays in the 125 μg g^-1^ K treatment (**Figure 2**). The greater uptake of Rb into low K treated plants is likely a consequence of the high background K present in plants receiving adequate soil K which would be expected to both reduce Rb uptake and to dilute Rb concentrations in the larger biomass of the K replete plants.

### PLANT BIOMASS

Though differences were small and variability was high the responses of plant biomass to soil and foliar treatment were similar to those observed with shoot leaf area and shoot length. Plant biomass (trunk diameter, 1- and 2-years-old shoot mass) was significantly greater in all high soil K treatments than low K soil treatments (**Table 2**). Under low K conditions foliar treatments had a small positive effect on trunk diameter, 1- and 2-years-old branches with the Bio-1 treatment having greatest diameter and mass which is consistent with results observed in shoot leaf area. Under high soil K conditions, Bio-2 had the highest diameter (equivalent to control) and mass of 2-years-old branches, which is also consistent with the patterns observed in shoot leaf area. The biostimulant effects were clearer when one and 2-years-old shoots were added together. Under low soil K conditions, Bio-1 and Bio-2 had an increment of 26 and 13% against control plants. However, only Bio-1 was declared to be significantly different from control plants. Even though positive trends were clear, significant differences were often not detected. A statistical power analysis revealed that the likelihood of having a Type II error (false negatives) was very high (**Table 2**).

**Table 2:** Tree harvest.

Potassium level (μg g^--1^)	Foliar treatment	Trunk diameter (cm)					1-year-old Branches (g)				2-years-old branches (g)			Sum of branches (g)			
		LS mean	Letter				LS Mean	Letter			LS mean	Letter		LS mean	Letter		
124	Foliar – K	11	a				285	a	b		156	a	b	441	a		
124	Foliar – (Bio-2)	11	a				296	a			194	a		490	a		
124	Foliar – (Bio-1)	10.6	a	b			323	a			183	a		506	a		
124	Foliar – Control	10.6	a	b			338	a			127	a	b	465	a		
5	Foliar – (Bio-1)	9.3		b	c		208		b	c	145	a	b	353	a	b	
5	Foliar - Control	9			c	d	187			c	94		b	281			c
5	Foliar – K	8.4			c	d	171			c	110		b	281		b	c
5	Foliar – (Bio-2)	8.4				d	213		b	c	105		b	318		b	c
Power analysis		0.38					0.35				0.15			0.1			

Multiple mean comparison was performed using Student’s *t-*test. Values within a column that have a different letter differ significantly at an α level of 0.05 (*n* = 20). Treatments and letters were sorted accordingly to the trunk diameter results. The statistical power of the factorial design was included for all the response variables. Note that power values are very low and therefore there is a high likelihood of having a type II error (not detecting significant differences when there may be).

## DISCUSSION

Determining the mode of action of biostimulants that are derived from complex extractions and contain a largely undefined mixture of organic and inorganic constituents is a complex task fraught with the potential for misinterpretation and unrecognized interactions. In the experiments conducted here it was practically impossible to construct a control that isolated the effects of each of the numerous chemical constituents present in each of the spray mixes. It is also commonly asserted by manufacturers of biostimulants that the beneficial effect of the biostimulant in question is dependent upon the combined and synergistic interaction of all components and hence experimentation conducted utilizing partial formulations would be unrepresentative. Recognizing these constraints the goal of this experiment was to provide a rigorous examination of the effects of these materials on plant growth attributes and on the uptake of potassium (utilizing Rb^+^ as a surrogate) in a tree species under stressed (K) and non-stressed conditions.

When K was provided in abundance both the foliar biostimulant 1 (MegaFol), derived from *A. nodosum* with additional plant based amino acids, betaines and vitamins and biostimulant 2 (GroZyme), derived from microbial fermentation extracts had a profound effect on shoot leaf area and number of leaves, while only biostimulant 2 significantly enhanced shoot length. Both biostimulants also increased average leaf size. The extent of growth stimulation by both biostimulants 1 and 2 was quite remarkable with a near doubling of shoot leaf area when plants were grown under what the authors experience suggests would be near ideal conditions. Visual observations indicated that both the biostimulants 1 and particularly the biostimulant 2 treatments resulted in plants that were unusually luxuriant in their growth, exceeding any response that the authors would expect from a foliar nutrient application alone.

When K was deficient the stimulatory effect of biostimulant 2 on shoot leaf area and number of leaves was not observed. In contrast biostimulant 1, which is known to stimulate stress response mechanisms (Petrozza et al., 2014) was effective at enhancing shoot and leaf growth though not to the extent observed under adequate K nutrition. Foliar K applications, which would be expected to partially mitigate a soil K deficit, were as effective as biostimulant 1 in promoting plant growth but neither treatment resulted in plant growth equivalent to plants provided with adequate K. While it is attractive to ascribe the positive effect of biostimulant 1 to its ability to mitigate K deficiency induced stress, an alternate explanation is that biostimulant 2, which contains equivalent K concentrations to the foliar K treatment, was merely acting as a foliar K fertilizer. The application of the foliar K fertilizer, which only occurred after sample date 2 in year 2, resulted in an immediate increment in plant growth equivalent to the biostimulant 1 applications. In this context, however, the failure of biostimulant 2 to recover plant growth under the low K treatment is puzzling since biostimulant 2 contained a highly effective K source in amounts greater than either the K foliar or the biostimulant 1 products. The mode of action of biostimulant 1 thus remains unclear.

Further evidence that plant response to biostimulant 1 application was not a consequence of the provision of foliar K is provided by the results of the Rb^+^ uptake. Potassium uptake is known to be tightly regulated by internal K concentrations uptake and the principle root K^+^ transporters (Shaker K^+^ channel AKT1 and KUP/HAK/KT transporter HAK5) are clearly regulated, both directly and indirectly, by internal K concentrations (Wang and Wu, 2013). Rubidium has been successfully used as a surrogate for K uptake in short-term experiments (Restrepo-Diaz et al., 2008; Pyo et al., 2010; Winkler and Zotz, 2010; Benlloch-Gonzalez et al., 2012), hence, the greatly increased Rb^+^ uptake into K^+^ starved roots observed here, is consistent with the principle that low internal [K] enhances root K^+^ (Rb^+^) uptake. The application of foliar K^+^ or Rb^+^ would not therefore be expected to enhance root K^+^ (Rb^+^) uptake (Benlloch-Gonzalez et al., 2012) a result that was supported by the observation in these experiments that the K foliar treatment did not enhance Rb^+^ uptake. In a similar experiment, Restrepo-Diaz et al. (2008) showed that olive plants that were K deficient had a lower foliar uptake of K (using Rb as analog) than plants that had adequate K concentrations. This suggests that nutrient deficient plants may have a lower capacity to take up foliars, which may explain the lack of differences observed in the foliar K^+^ treatment, but not the benefits observed in the biostimulant treatments. The significant enhancement of Rb^+^ uptake by both biostimulants 1 and 2 under low K supply and the enhancement of Rb^+^ uptake by biostimulant 1 under high K soil treatments cannot therefore be explained by their K^+^ content.

Biostimulant 1 was clearly designed to contain a number of molecules known to be active in plant stress response pathways (Mittler, 2002, 2006; Zhu, 2002; Delorge et al., 2014; Petrozza et al., 2014) and evidence suggests it is effective in stimulating plant stress response genes (Petrozza et al., 2014). While this mechanism is supported by the observation that biostimulant 1 enhanced plant growth under K stress, the apparent benefits of biostimulant 1 under non-stress conditions suggests either that other growth stimulatory effects exist or that the K replete plants were suffering from an unknown stress at some time in their growth. Since plants were grown in an outdoor location and as temperatures in summer in Davis, CA, USA frequently exceed 38°C and water stress may have occurred for brief periods between irrigations, it is plausible that short duration plant stress did in fact occur.

Biostimulant 2 is a microbial fermentation utilizing bacteria that includes plant growth promoting rhizobacteria (PGPR) isolates. The molecules that are generated from the fermentation and subsequent isolation and purification process have not been characterized. PGPR have a wide range of effects on plants including enhancing biotic and abiotic stress resistance and increasing plant nutrient uptake (Calvo et al., 2014). To our knowledge there has been no investigation of the effect of PGPR or their metabolites when applied as a foliar spray. The plant responses to biostimulant 2 observed here, which included marked leaf, and shoot expansion were expressed only under optimal (K^+^) growth conditions a result that does not suggest alleviation of a nutrient deficiency but is more suggestive of a true biostimulation. Biostimulant 2 is not known to contain significant quantities of any identified plant hormone.

Biostimulant 2 does, however, include N, P, K, S, Cu, Zn, Fe, Mn and hence, its positive effect may suggest that a sub-clinical deficiency of one or more of these elements may have been present and that positive effects of biostimulant 2 were merely due to correction of a nutrient deficiency. In a companion paper in this issue (Tian et al., 2015), foliar application of biostimulant 2 was shown to positively increase Zn uptake and within plant transport. Significantly, the addition of a single subcomponent of biostimulant 2 (GroZyme) which does not contain Zn was effective in enhancing within plant Zn mobility (Tian et al., 2015). While the possibility that biostimulant 2 acted solely to alleviate an unrecognized nutrient deficiency cannot be dismissed this is considered unlikely since plants were provided with a complete nutrient solution and no visual sign of a nutrient deficiency was evident. Further, the growth responses to biostimulant 2, which included a 195% increase in shoot leaf area, were not consistent with alleviation of a sub-clinical nutrient deficiency.

The results presented here show significant plant growth benefits of two biostimulants of diverse origin and also highlight the complexity of research utilizing complex mixes of poorly defined metabolites. The mode of action of the products utilized here remains unresolved though biostimulant 1 does not appear to function solely through its role in stress mitigation while biostimulant 2 may influence plant growth through both mitigation of plant stress and stimulation of plant growth in non-stress conditions. Biostimulants are defined as substances or material, with the exception of nutrients and pesticides, which have the capacity to beneficially modify plant growth. Significantly, this definition does require that the mode of action is understood a condition that greatly compromises the development of products that can be used with consistent and predictable effect.

## Conflict of Interest Statement

A portion of the funding for this research was obtained from Valagro and from Ag Spectrum, manufacturers of the two biostimulants used in this experiment.
